# Supplementary material for: Molecular mechanisms underlying the BIRC6-mediated regulation of apoptosis and autophagy
Source: Nat Commun. 2024 Jan 30;15:891. doi: 10.1038/s41467-024-45222-1 (PMC10827748; doi:10.1038/s41467-024-45222-1)
Supplement: Supplementary file 1 — Supplementary Information [file 41467_2024_45222_MOESM1_ESM.pdf]

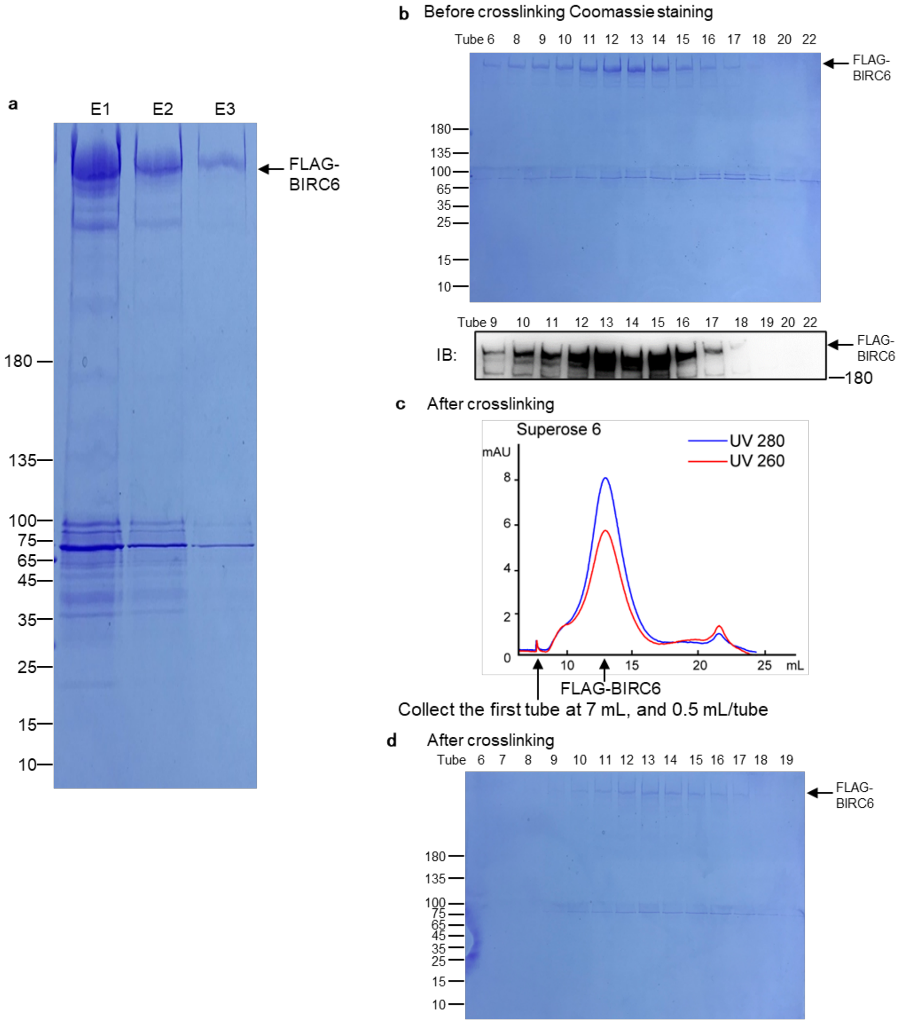

e

Number	Protein	Accession	Description	Score	Coverage [%]	# Peptides	MW [kDa]
1-2	BIRC6	Q9NR09	Baculoviral IAP repeat-containing protein 6 OS=Homo sapiens GN=BIRC6	2272.42	57	222	529.9
32-8	UBA6	A0AVT1	Ubiquitin-like modifier-activating enzyme 6 OS=Homo sapiens GN=UBA6	321.53	61	48	117.9
31-178	Nrdp1	Q9H4P4	E3 ubiquitin-protein ligase NRDP1 OS=Homo sapiens GN=RNF41	55.01	38	8	35.9
32-1219	HtrA2	O43464	Serine protease HTRA2, mitochondrial OS=Homo sapiens GN=HTRA2	5.77	2	1	48.8
4--10	Smac	Q9NR28	Diablo homolog, mitochondrial OS=Homo sapiens GN=DIABLO	157.62	67	15	27.1
4-342	Casp14	P31944	Caspase-14 OS=Homo sapiens GN=CASP14	13.21	19	5	27.7
4-360	Casp3	P42574	Caspase-3 OS=Homo sapiens GN=CASP3	12.11	19	5	31.6
4-380	Casp6	P55212	Caspase-6 OS=Homo sapiens GN=CASP6	11.18	10	3	33.3
4-528	Casp7	P55210	Caspase-7 OS=Homo sapiens GN=CASP7	6.39	7	2	34.3
4-830	LC3B2	A6NCE7	Microtubule-associated proteins 1A/1B light chain 3 beta 2 OS=Homo sapiens GN=MAP1LC3B2	2.31	11	1	14.6

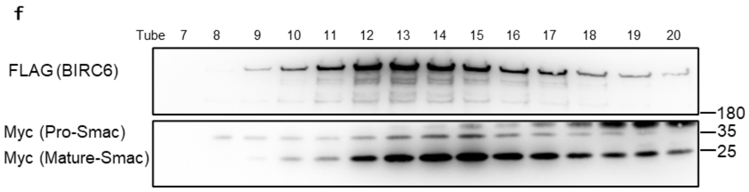

Supplementary Fig. 1 Purification of the FLAG-BIRC6 from HEK293F cells.
a 0.5 mg/mL FLAG peptide in PBS supplemented with 100 mM KCl was used to elute FLAG-BIRC6 at 4°C for 30 min, and repeated for 3 times. Protein samples were analyzed by Coomassie staining following SDS-PAGE. **b** The elution fraction was passed through the gel filtration-Superose 6 Increase Columns for the first time. The fraction at 0.5 mL/tube was collected starting from 7 mL. The tubes 6, 8-18, 20, 22 were analyzed by Coomassie staining following SDS-PAGE. The tubes 9-20 and 22 were analyzed by immunoblotting following SDS-PAGE. **c** The peak elution tubes 11-15 from (**b**) were combined for crosslinking by 0.01% glutaraldehyde, and passed through the Superose 6 Increase Columns once more. **d** The elutes from tubes 6-19 in (**c**) were analyzed by Coomassie following SDS-PAGE. **e** The purified FLAG-BIRC6 complex was analyzed by mass spectrometry following separation of the complex by SDS-PAGE. The full lane was sliced into 6 pieces from top to bottom with assigned sample numbers: 1, 2-1, 2-2, 3-1, 3-2, and 4. Potential BIRC6-interacting proteins are listed, and others are provided in online materials. **f** 0.5 mg/mL FLAG peptide in PBS supplemented with 100 mM KCl was employed to elute FLAG-BIRC6-Myc-Smac complex at 4°C for 30 min, and repeated for 3 times. After gel filtration, the peak fractions tubes7-20 were analyzed by immunoblotting following SDS-PAGE. Source data for (**a**), (**b**), (**d**), and (**f**) are provided as a Source Data file.

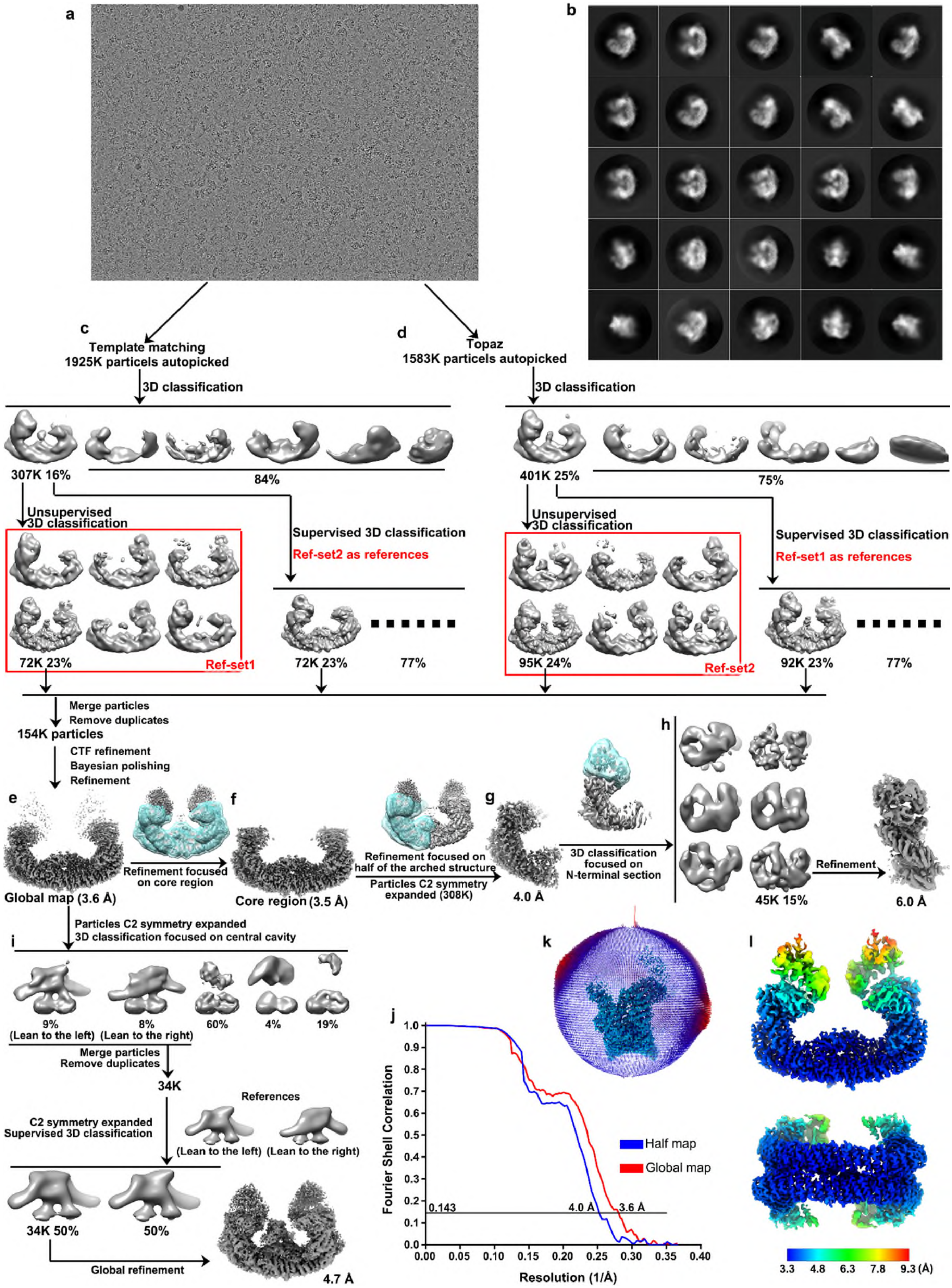

a
b
c
Template matching
1925K particels autopicked
3D classification
307K 16%
84%
Unsupervised
3D classification
72K 23%
Ref-set1
Supervised 3D classification
Ref-set2 as references
72K 23%
77%
d
Topaz
1583K particels autopicked
3D classification
401K 25%
75%
Unsupervised
3D classification
95K 24%
Ref-set2
Supervised 3D classification
Ref-set1 as references
92K 23%
77%
Merge particles
Remove duplicates
154K particles
CTF refinement
Bayesian polishing
Refinement
e
Global map (3.6 Å)
Refinement focused
on core region
f
Core region (3.5 Å)
Refinement focused on
half of the arched structure
Particles C2 symmetry
expanded (308K)
g
4.0 Å
3D classification
focused on
N-terminal section
h
45K 15%
Refinement
6.0 Å
Particles C2 symmetry expanded
3D classification focused on central cavity
i
9%
(Lean to the left)
8%
(Lean to the right)
60%
4%
19%
Merge particles
Remove duplicates
34K
References
C2 symmetry expanded
Supervised 3D classification
(Lean to the left)
(Lean to the right)
34K 50%
50%
Global refinement
4.7 Å
j
Fourier Shell Correlation
Resolution (1/Å)
0.143
4.0 Å
3.6 Å
Half map
Global map
k
l
3.3
4.8
6.3
7.8
9.3 (Å)

Supplementary Fig. 2 Cryo-EM image processing workflow of BIRC6 sample.

a Representative Cryo-EM image of BIRC6 sample. **b** 2D class averages of BIRC6. **c-d** Preliminary 3D classifications of particles autopicked using template matching method (**c**) or autopicked using Topaz (**d**). **e** The good particles from (**c-d**) were merged and subjected to 3D refinement, generating a final global density map at a resolution of 3.6 Å. **f** Refinement focused on the core region. **g** Local refinement focused on half of the U-shape structure. **h** 3D classification focused on N-terminal session. **i** 3D classification focused on the residual density in the central cavity. **j** Fourier shell curves from the refinements of global map (**e**) or half of the U-shape map (**g**). **k** Euler angular distribution of the particles in the last round of structural refinement of the global map. **l** Local resolution heat map of the composite density map displayed in two different views.

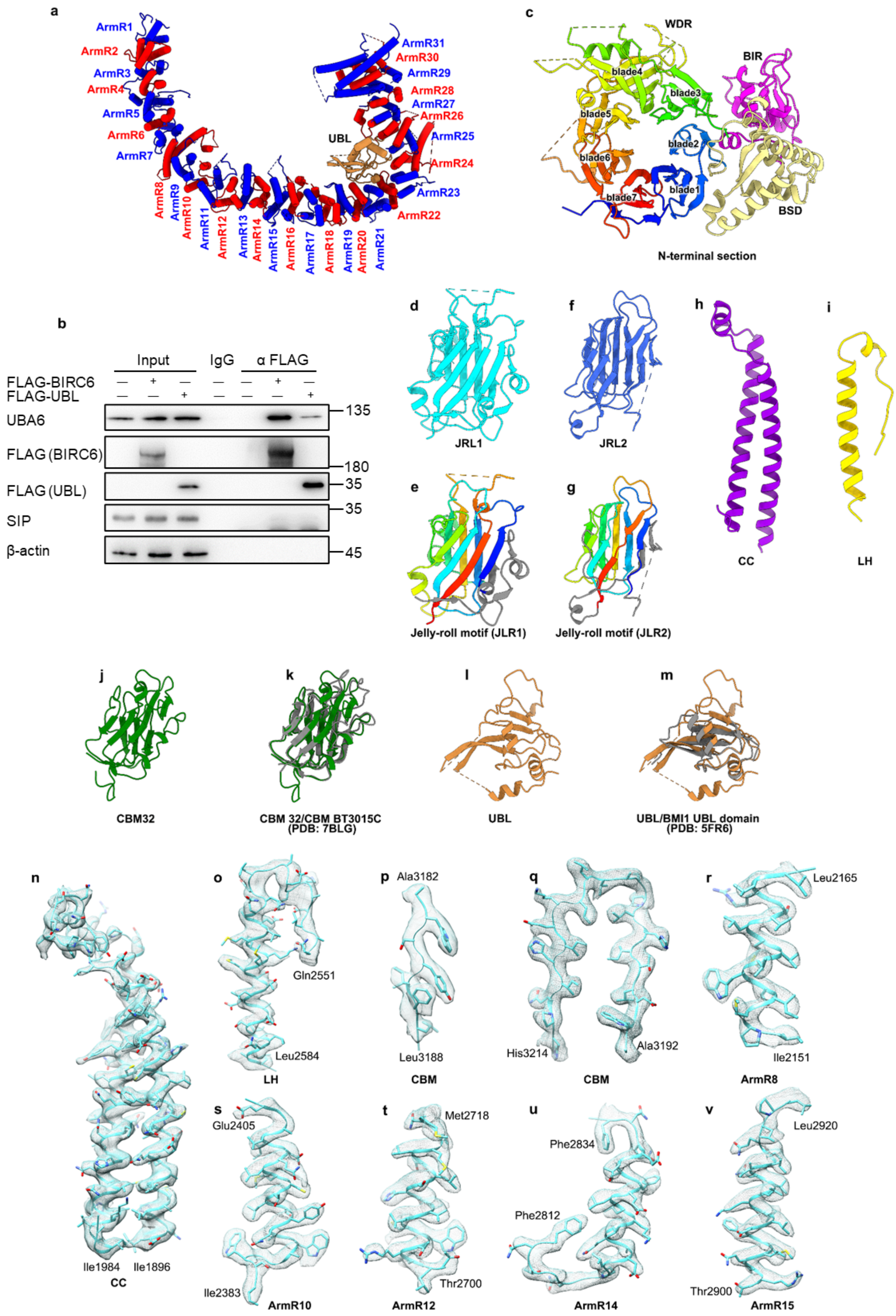
a
ArmR1
ArmR2
ArmR3
ArmR4
ArmR5
ArmR6
ArmR7
ArmR8
ArmR9
ArmR10
ArmR11
ArmR12
ArmR13
ArmR14
ArmR15
ArmR16
ArmR17
ArmR18
ArmR19
ArmR20
ArmR21
ArmR22
ArmR23
ArmR24
ArmR25
ArmR26
ArmR27
ArmR28
ArmR29
ArmR30
ArmR31
UBL
c
WDR
BIR
blade1
blade2
blade3
blade4
blade5
blade6
blade7
BSD
N-terminal section
b
Input
IgG
α FLAG
FLAG-BIRC6
FLAG-UBL
UBA6
FLAG (BIRC6)
FLAG (UBL)
SIP
β-actin
135
180
35
35
45
d
JRL1
e
Jelly-roll motif (JLR1)
f
JRL2
g
Jelly-roll motif (JLR2)
h
CC
i
LH
j
CBM32
k
CBM 32/CBM BT3015C (PDB: 7BLG)
l
UBL
m
UBL/BMI1 UBL domain (PDB: 5FR6)
n
Ile1984
Ile1896
CC
o
Gln2551
Leu2584
LH
p
Ala3182
Leu3188
CBM
q
His3214
Ala3192
CBM
r
Leu2165
Ile2151
ArmR8
s
Glu2405
Ile2383
ArmR10
t
Met2718
Thr2700
ArmR12
u
Phe2834
Phe2812
ArmR14
v
Leu2920
Thr2900
ArmR15

Supplementary Fig. 3 Structures of BIRC6 domains.
a Structure of ArmRD with a sharp bending around ArmR21-24. The 31 ArmRs are colored blue and red alternatively. UBL domain located at the bending site is also shown and labeled. **b** HEK293T cells were transfected with FLAG-BIRC6 and the FLAG-tagged UBL (the ubiquitin-like domain of BIRC6: residues 3806-4054). Immunoblotting was performed following immunoprecipitation with anti-FLAG antibodies. **c** Structure of the N-terminal region. WDR is colored rainbow and the seven blades are labeled. BIR and BSD are colored-colored as that in (**Fig. 1a, c) d-e** Structure of JRL1. (**d**) Same as (**e**) but with the jelly-roll motif colored rainbow and the left sequences colored gray. **f-g** Similar to (**d, e**) but showing the structure of JRL2. **h** Structure of CC. **i** Structure of LH. **j** Structure of CBM32. **k** Same as (**j**) but with another CBM32 structure CBM BT3015C (gray, PDB: 7BLG) superimposed. **l** Structure of UBL. **m** Same as (**l**) but with UBL domain of BMI1 (B cell-specific Moloney murine leukemia virus integration site 1) (gray, PDB: 5FR6[27]) superimposed. **n-v** Representative local density maps in the key structural elements. Source data for (**b**) are provided as a Source Data file.

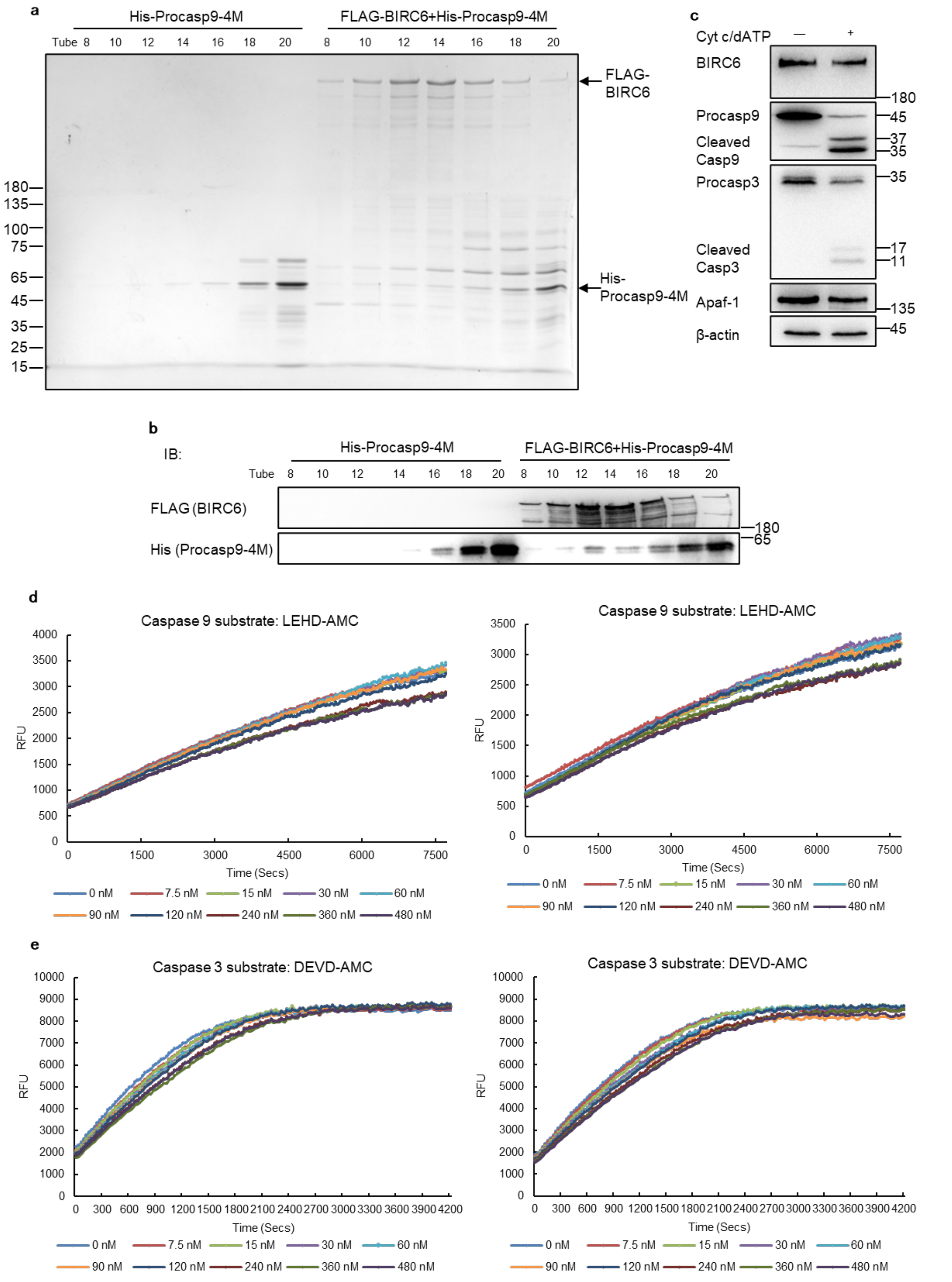

Supplementary Fig. 4 Purified BIRC6 strongly inhibits the activity of caspase 9, but only weakly for caspase 3.
a, b About 0.5 mg His-procasp9-4M was added to 1.2 mL of FLAG-BIRC6 at 0.03 μM in elution buffer, and incubated 30 min at room temperature. The reaction system was passed through a Superose 6 Column equilibrated with PBS plus 100 mM KCl. The fraction at 0.5 mL/tube was collected starting from 7 mL, and tubes 8, 10, 12, 14, 16, 18, 20 were collected for Coomassie staining (**a**) or immunoblotting (**b**) following SDS-PAGE. His-Casp9-4M alone was passed through Superose 6 Column, which was the control in the absence of BIRC6. **c** Caspases in HEK293T cell extracts were activated by cytochrome c and dATP, and protein levels were analyzed by immunoblotting. **d, e** Active caspases in HEK293T cell extracts were incubated with the purified FLAG-tagged BIRC6 at indicated concentrations. The caspase activities were analyzed in duplicates using the caspase 9 substrate Ac-LEHD-AMC (**d**) or caspase 3 substrate Z-DEVD-7-AMC (**e**). The release of AMC from the substrates was monitored continuously at 380/460 nm (excitation/emission) at 30°C for up to 2 h. Source data for (**a**- **c**) are provided as a Source Data file.

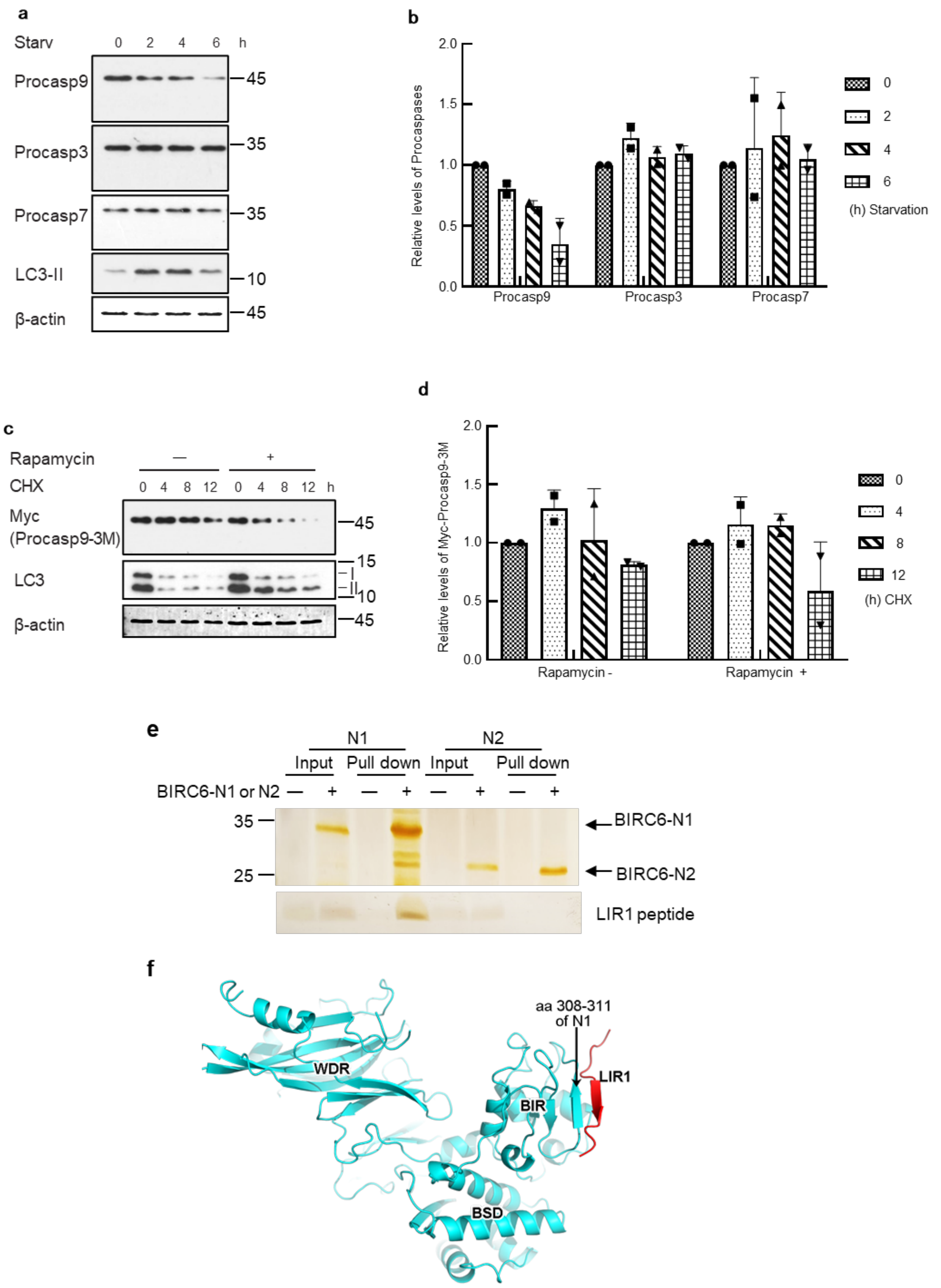

Supplementary Fig. 5 Degradation of Procaspase 9 by autophagy mediated by LC3.

a, b MDA-MB-453 cells were starved in HBSS for 0, 2, 4, or 6 h. The protein levels were detected by immunoblotting (**a**), and procasp9, 3, or 7 was quantified by densitometry (normalized to β-actin) with two independent biological replicates. (**b**). **c, d** HEK293T cells were transfected with Myc-tagged procaspase 9 cleavage site mutant (Procasp9-3M) for 24 h, and then treated with 0 or 0.5 μM rapamycin. After rapamycin treatment for 24 h, 50 μg/mL CHX was added and incubated for the indicated periods of time. The protein levels were detected by immunoblotting (**c**), and quantified by densitometry (normalized to β-actin) (**d**). Data are representative of one experiment with two independent biological replicates. **e** 0.5 mg LIR1 peptides (NPQTSSFLQVLV) were incubated with His-tagged BIRC6-N1 (aa 27-358) or BIRC6-N2 (aa 358-543) for 30 min at RT. Silver staining was performed following the pulldown assay by Ni-NTA and SDS-PAGE. Data are representative of one experiment with three independent biological replicates. **f** The predicted interaction between the LIR1 peptide and the N-terminal section of BIRC6. The N-terminal section and LIR1 are colored cyan and red, respectively. Source data for (**a-e**) are provided as a Source Data file.

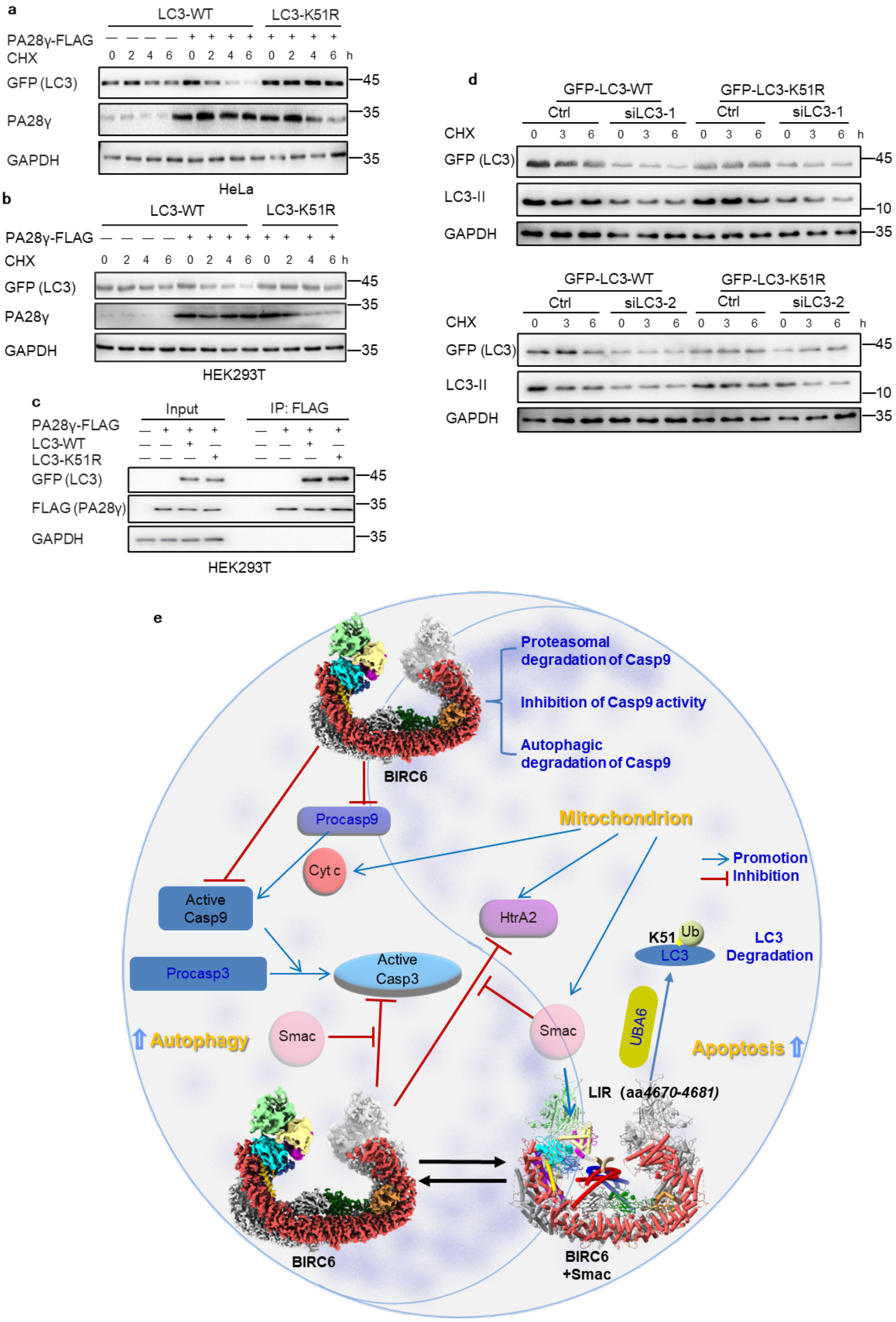

Supplementary Fig. 6 K51 mutation of LC3 promotes autophagy, but suppresses apoptosis.

a, b HeLa cells (**a**) or HEK293T (**b**) cells were co-transfected with GFP-LC3-WT or LC3-K51R and PA28γ-FLAG, and then treated with 100 μg/mL CHX for the indicated periods of time. Protein levels were analyzed by immunoblotting with two independent biological replicates. **c** HEK293T cells were co-transfected with GFP-LC3-WT or LC3-K51R and PA28γ-FLAG. Protein levels were analyzed by immunoblotting following co-immunoprecipitation using the anti-FLAG antibody. **d** HEK293T cells were transfected with GFP-LC3-WT or LC3-K51R and LC3 siRNA1 (GAGUGAGAAAGAUGAAGAUTT) or siRNA2 (CGUCGGAGAAGACCUUCAATT), and then treated with 100 μg/mL CHX for 0, 3, or 6 h. Protein levels were analyzed by immunoblotting with two independent biological replicates. **e** Model mechanisms by which BIRC6 balances apoptosis and autophagy. A background of Taiji diagram hints the nature of the balance between apoptosis and autophagy. Source data for (**a-d**) are provided as a Source Data file.

Supplementary Table 1 Cryo-EM data collection, refinement and validation statistics of samples

	Global map of BIRC6 (EMD-35759) (PDB: 8IVQ)	Composite map (EMD-38464)	Core region of BIRC6 (EMD-38461)	Half map of the core region (EMD-38462)	N-terminal region (EMD-35758)	BIRC6-Smac complex (EMD-35760)
**Data collection and processing**						
Nominal magnification			64,000×			
Voltage (kV)			300			
Electron exposure ($e^{-}/Å^{2}$)			68.4			
Defocus range (μm)			-2 to -3			
Pixel size (Å)			1.37			
Micrographs			7331			
Symmetry imposed	C2	C2	C2	C1	C1	C1
Initial particle images			1925K/1583K			
Final particle images	154K	154K	154K	308K	45K	34K
Map resolution (Å)	3.6	3.5	3.5	4.0	6.0	4.7
FSC threshold	0.143	0.143	0.143	0.143	0.143	0.143
**Refinement**						
Model composition						
Non-hydrogen atoms	41900					
Protein residues	5842					
R.m.s. deviations						
Bond lengths (Å)	0.0048					
Bond angles (°)	1.00					
Validation						
MolProbity score	1.26					
Clashscore	5.02					
Poor rotamers (%)	0					
Ramachandran plot						
Favored (%)	98.08					
Allowed (%)	1.92					
Disallowed (%)	0					

Supplementary Table 2 List of primer pairs used in experiments

Cloning vectors	Primers
pMlink-3×Flag-UBL (EcoRI/XhoI)	F: ATATGAATTCATGGAGAAGGTGACAATGTTTCTTCAGTCACC<br>R: ATATCTCGAGTTAAATAGGACAGGTTTCCAGCAAAGATTCATCCAATAC
pET-30a-His-procaspase 9-4M (BamHI/NotI)	F: ATATGGATCC ATGGACGAAGCGGATCGGCGGCT<br>R: ATATGCGGCCGC GACATCACCAAATCCTCCAGAACCAATGT
pET-28a-His-BIRC6-N1 (BamHI/NotI)	F: ATATGGATCCGTCTCCGAGTGGCTGGTGCTGCGGG<br>R: ATATGCGGCCGCTCAATCTGCAGAAGGGAGCTGCGC
pET-28a-His-BIRC6-N2 (BamHI/NotI)	F: ATATGGATCCGATGGAGCTGACAGAATAGCTTGCTTTG<br>R: ATATGCGGCCGCTCACAATAAACAGGGGAAGGGAATGTTG
