# Supplementary material for: Molecular mechanisms underlying the BIRC6-mediated regulation of apoptosis and autophagy
Source: Nat Commun. 2024 Jan 30;15:891. doi: 10.1038/s41467-024-45222-1 (PMC10827748; doi:10.1038/s41467-024-45222-1)
Supplement: Supplementary file 3 — Description of Additional Supplementary Files [file 41467_2024_45222_MOESM3_ESM.pdf]

Description of Additional Supplementary Files

File Name: Supplementary Data 1
Description:

List of all Mass Spectrometric Data.
